# Fibroinflammatory Diseases of Aorta: The Inside Look

**DOI:** 10.1055/a-2781-8133

**Published:** 2026-01-27

**Authors:** Rabin Gerrah

**Affiliations:** 1 Department of Cardiothoracic Surgery, Stanford University Cardiovascular Institute, Stanford University, Stanford, California, United States

**Keywords:** Erdheim Chester disease, fibroinflammatory disease, aorta

## Abstract

Erdheim Chester disease (ECD) is a rare fibroinflammatory disease that affects different segments of aorta. It appears as diffuse wall thickening and periaortic accumulation of scar tissue on computerized tomography (CT). The CT scans describe the size and the external structure, with minimal description of endoluminal surface of the aorta. In this report, CT images were used to visualize the interior surface of the aorta and the response to treatment in a patient with ECD.

## Introduction

Fibroinflammatory diseases (FID) affecting the aorta are rare entities with significant diagnostic challenge. Involvement of the aortic wall, in the inflammatory process renders an imaging finding of irregular aortic wall, which might raise the suspicion for intramural hematoma or acute aortic dissection. The disease manifestation depends on the layer predominantly involved in the inflammatory process: while inflammation of the inner layers will potentially cause luminal constriction, outer layer results in degeneration, and development of aneurysms. The two typical FIDs involving the thoracic aorta are Erdheim Chester disease 1 and IgG4-related disease. 2

While the computerized tomography (CT) remains the gold standard imaging method for FID, it focusses mostly on the size and external structure of the aorta, showing the nonspecific findings of diffuse wall thickening and periaortic accumulation in FIDs. Furthermore, it is never used to provide an internal view inside the aorta.

## Case Presentation

**Fig. 1:**
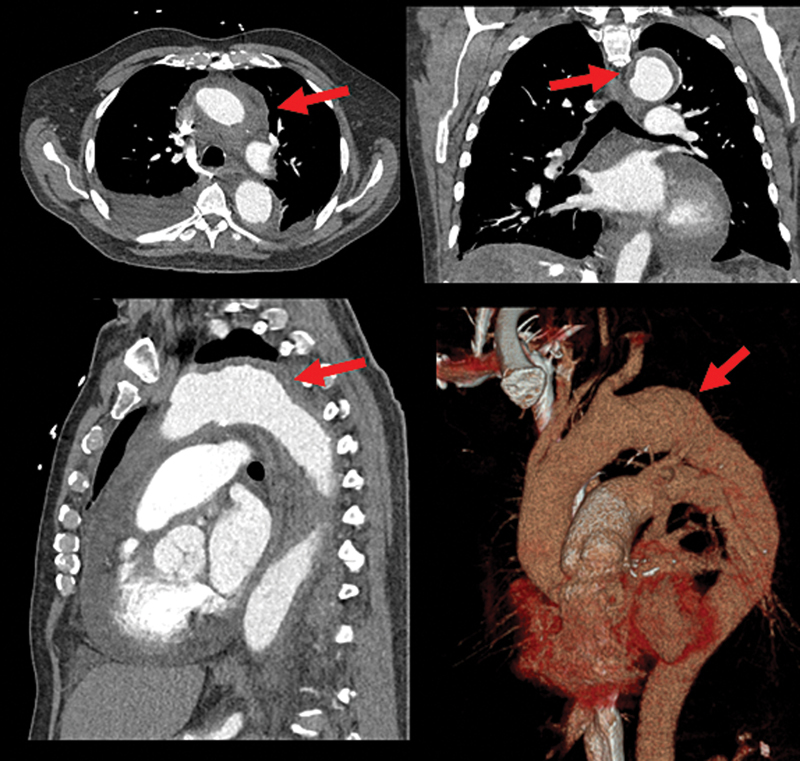
Standard computerized tomography (CT) images, all showing the irregular aortic wall and deposition in periaortic region (arrow).

In this report, CT images of a patient suspected for Erdheim Chester disease ( Fig. 1 ) were further analyzed by a previously described technique 3 for intraluminal visualization. In this technique, the CT images (as DICOM files) are imported to InVesalius 3.1.1 (Renato Archer Information Technology Center) and automatically segmented to reconstruct patient-specific 3D meshes of the region of interest. The resulting 3D meshes are saved as .stl files. The .stl files are then imported into Meshmixer 3.5 (Autodesk Inc) for 3D visualization and manipulation that enables intraluminal visualization. This 73-year-old man presented to emergency department with chest pain and shortness of breath. He underwent initial evaluation that included cross-sectional imaging of the chest. Initial chest CT scan showed enlarged aorta with significant irregularity throughout the aorta. The irregularity of the aortic wall raised the suspicion for FID involving aorta; however, additional findings such as pericardial and pleural effusions took priority in patient care, both treated with pericardiocentesis and thoracentesis, respectively. After extensive immunological and additional imaging that was typical with periaortic tissue infiltration, 4 the final diagnosis was confirmed as Erdheim Chester disease.

**Fig. 2:**
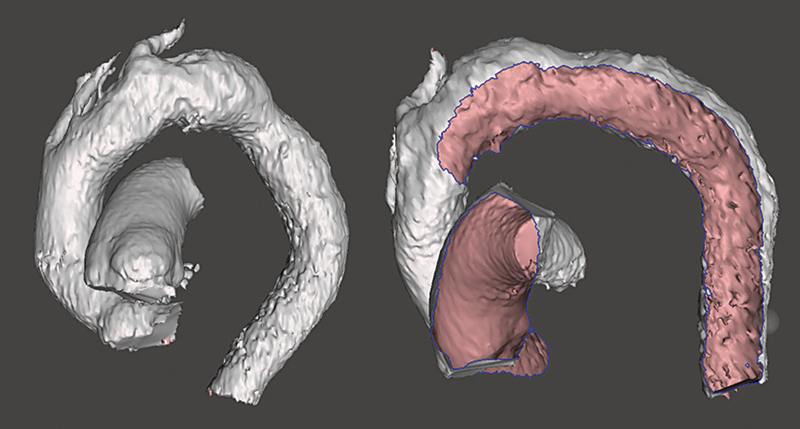
Three-dimensional imaging of the aorta and pulmonary artery in fibroinflammatory disease with endoluminal visualization. Note the difference in the disease involvement on interior surface of the aorta compared with pulmonary artery. Sections of the pulmonary artery are removed to simplify the visualization. Since Erdheim Chester disease involve aorta more than pulmonary artery, the endoluminal surface of the pulmonary artery shows a smoother surface compared with the endoluminal surface of aorta. This irregularity corresponds to infiltration in the aortic wall that assists in diagnosis of Erdheim Chester disease.

**Fig. 3:**
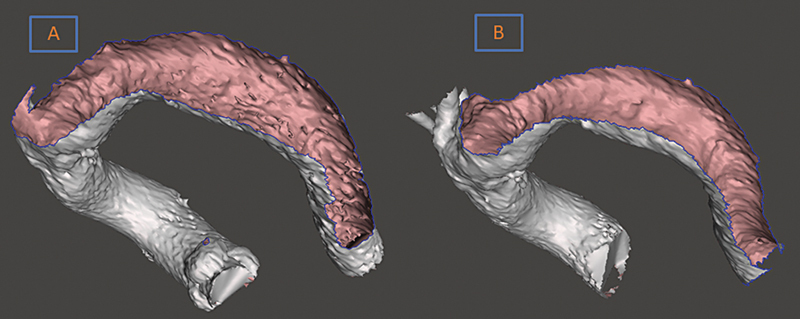
Endoluminal visualization of the aorta at the time of initial diagnosis (A) and after a short course (3 weeks) of corticosteroid treatment (prednisone 60 mg/d) (B). After treatment with corticosteroids, the endoluminal surface has become smoother due to partial resolution of the infiltration.

The intraluminal visualization provided invaluable information on the extent of the inner aortic layer involvement, ( Fig. 2A ), as well as endoluminal view of a noninvolved large vessel (pulmonary artery) for comparison ( Fig. 2B ), and even the response to 3 weeks of treatment with steroids ( Fig. 3A, B ). This visualization reveals that similar irregularity that was seen on outside surface of the vessel, presents on interior surface as well, a fact that has been rarely described by CT image readings. Whether these irregularities have a clinical impact, such a predilection for thrombogenesis, or embolic events is unclear. Of more concern for surgical consideration, it is unclear how would the aortic tissue react to surgical intervention of the aorta with this underlying condition, if such an intervention was needed.

Treatment for Erdheim Chester disease includes targeted therapy such as Vemurafenib, immunotherapy with Interferon alpha, and chemotherapy as well as steroids. Our patient was treated with steroids with good initial response. Surgical treatment for aorta involvement has not reported due to inflammatory process of the disease.

## Discussion

Intraluminal visualization of the aorta reveal unique characteristics often ignored in routine CT imaging an adjunct to the CT scans. The internal surface of aorta clearly visualized in disease affected and nonaffected vessels as well as the changes with the treatment.

Whether these interior images of the aorta obtained from the CT scans can differentiate between different aortic pathologies is unknown. It is also unclear whether this visualization can provide any prognostic value or a prediction in aortopathy processes. However, in pathologies such as FID, as other confirmatory modalities are limited and tissue sampling with biopsy is not practical, this endoluminal visualization is potential adjunct to support the diagnosis of this pathology.
